# Preservation Mechanism of Chitosan-Based Coating with Cinnamon Oil for Fruits Storage Based on Sensor Data

**DOI:** 10.3390/s16071111

**Published:** 2016-07-18

**Authors:** Yage Xing, Qinglian Xu, Simon X. Yang, Cunkun Chen, Yong Tang, Shumin Sun, Liang Zhang, Zhenming Che, Xihong Li

**Affiliations:** 1 Sichuan Province Key Laboratory of Grain and Oil Processing and Food Safety, Food and Bioengineering College, Xihua University, Chengdu 610039, China; Tangbohu3@163.com (Y.T.); ssmin2000@163.com (S.S.); zhangliang1872@163.com (L.Z.); chezhenming@163.com (Z.C.); 2 School of Engineering, University of Guelph, Guelph, ON N1G 2W1, Canada; syang@uoguelph.ca; 3 Key Laboratory of Physiological and Storage of Agricultural Products after Harvest in the Ministry of Agriculture, National Engineering Technology Research Center for Preservation of Agricultural Products, Tianjin 300384, China; cck0318@126.com; 4 Food Engineering and Biotechnology College, Tianjin University of Science & Technology, Tianjin 300457, China; lixihong6061@aliyun.com

**Keywords:** preservation mechanism, chitosan coating, cinnamon oil, sensor data

## Abstract

The chitosan-based coating with antimicrobial agent has been developed recently to control the decay of fruits. However, its fresh keeping and antimicrobial mechanism is still not very clear. The preservation mechanism of chitosan coating with cinnamon oil for fruits storage is investigated in this paper. Results in the atomic force microscopy sensor images show that many micropores exist in the chitosan coating film. The roughness of coating film is affected by the concentration of chitosan. The antifungal activity of cinnamon oil should be mainly due to its main consistent trans-cinnamaldehyde, which is proportional to the trans-cinnamaldehyde concentration and improves with increasing the attachment time of oil. The exosmosis ratios of *Penicillium citrinum* and *Aspergillus flavus* could be enhanced by increasing the concentration of cinnamon oil. Morphological observation indicates that, compared to the normal cell, the wizened mycelium of *A. flavus* is observed around the inhibition zone, and the growth of spores is also inhibited. Moreover, the analysis of gas sensors indicate that the chitosan-oil coating could decrease the level of O_2_ and increase the level of CO_2_ in the package of cherry fruits, which also control the fruit decay. These results indicate that its preservation mechanism might be partly due to the micropores structure of coating film as a barrier for gas and a carrier for oil, and partly due to the activity of cinnamon oil on the cell disruption.

## 1. Introduction

Consumption of fruits with good quality might decrease the development risk of chronic diseases in human body and provide benefits for body health [1,2]. However, the losses of nutritional ingredients in fruits always occur because of its decay and physiological changes, which could be induced by the microbial infection during the storage time [3,4]. Recently, many researchers have developed semipermeable edible coatings as a carrier for antimicrobial agents in order to control the nutrient loss and maintain the quality of fruits [5,6,7,8]. This is because the edible coating could keep textural properties, sensory appearance and control respiration rate of fruits by functioning as a semipermeable vapor and gas barrier to regulate the moisture and gases in the package [5,9,10]. Moreover, the antimicrobial activity of edible coating could be significantly improved by incorporating different antimicrobial agents [3,4]. Thus, there is increasing interest in developing a bio-based coating with high antimicrobial activity for fruits storage, such as a chitosan coating with cinnamon oil [4,11,12].

Chitosan is recognized as safe and has been widely applied as a successful carrier for incorporating with the antimicrobial agents because of its adventurous biocompatibility and film-forming ability [2,6,12,13,14,15,16]. The preservation effect of chitosan-based coatings might be partly due to its property of slowing the respiration rate and controlling the decay of fruits, and partly due to its function of preventing the formation of off odors and retarding lipid oxidation in fruits [9,12,17,18]. Velickova et al. (2013) found that the chitosan-beeswax coating could slow down the respiration rate and reduce the senescence of strawberries [19]. As reported by Waewthongrak et al. (2015), the decay of citrus fruit could be significantly reduced by the combined application of chitosan and the crude extract from the culture medium of *Bacillus subtilis* [20]. Kaya et al. (2016) expressed that the application of chitosan and acetic acid could extend the shelf life of kiwifruit berries [21]. Moreover, Duran et al. (2016) indicated that the quality of strawberries could be maintained by chitosan coatings with nisin, natamycin, pomegranate and grape seed extract [22]. The incorporation of essential oils as the antimicrobial agent into the coating carrier is also considered as the successful technology to control the decay and maintain the quality of fruits during the storage time [4,23].

In the last few years, the addition of essential oil into the chitosan-based coating have been developed to enhance its antimicrobial property and act an active agent in the film coated on the surface of fruits [3,12,13,24,25]. Cinnamon oil as “Generally Recognized as Safe” by the Food and Drug Administration (FDA) has been recognized as the better antimicrobial activity because it is rich in cinnamaldehyde and/or trans-cinnamaldehyde [5,26,27,28]. As reported by several researchers, the after-harvested use of chitosan-based coating incorporating with cinnamon oil might control the postharvest fungi in pepper, prevent the growth of microorganisms in fresh cut pear and melon, and enhance the quality of jujube fruits [4,6,9,12,29,30,31]. According to the investigation of Carvalho et al. (2016), they reported the chitosan coating enriched with trans-cinnamaldehyde could improve the quality characteristics and extend the shelf life of melon slices to 15 days [29]. The enhancement on the antimicrobial activity of chitosan-based coating might be due to the addition of cinnamon oil or trans-cinnamaldehyde with good killing effect against bacteria and fungi [9,26]. 

For the antimicrobial mechanism of chitosan, its killing actions might be due to the interaction between chitosan molecules with positive charged and microbial cell membranes with negative charged and also due to the inhibited effectiveness on the microorganism growth and the toxins production [3,32,33,34,35]. However, the fresh keeping of chitosan-based coating and antifungal mechanism of cinnamon oil are still not very clear. Zhang et al. (2016) have investigated the mechanism of antibacterial action of cinnamon oil against *Escherichia coli* and *Staphylococcus aureus* by observing the changes of cell microstructure [36]. The investigation of Clemente et al. (2016) also investigated the antimicrobial properties and mode of action of cinnamon oil against foodborne bacteria [37]. In the series of papers published by our authors previously, the antimicrobial property and the application effectiveness of chitosan-based coating and cinnamon oil were mainly reported. Xing et al. (2010b) conducted the investigation on the in vitro and in vivo activities of cinnamon oil against three kinds of fungi [26]. Xing et al. (2010a) have extended the shelf life of lotus root slices with the chitosan-based solution [18]. Moreover, as conducted by Xing et al. (2011a), the in vivo control effects of chitosan-oil coating on the disease caused by *P. citrinum* of Lingwu long jujube fruits were evaluated [3]. Xu et al. (2013) have analyzed the effect of cinnamon oil fumigation and chitosan coating on the microbial safety of pear slices [6]. Xing et al. (2011b) and Xing et al. (2015) have investigated the effects of chitosan-oil coating on the activities of defense-related enzymes and the quality attributes of pepper and jujube fruits, respectively [4,9]. Nevertheless, less literature has been published by researchers about the keeping-fresh mechanism of chitosan-based coatings and the antifungal mechanism of cinnamon oil for the application effectiveness on the quality of fruits. 

Therefore, the objective in this article is to understand the preservation mechanism of chitosan-based coating and cinnamon oil based on sensor data. Firstly, the morphology and roughness analysis of coating film with different concentration of chitosan were conducted by AFM (atomic force microscopy) sensor. This section is mainly to understand its microstructure and ability as the modification of gas and as the carrier of antimicrobial agents. Secondly, the antifungal mechanism of cinnamon oil is investigated through the activity comparison between trans-cinnamaldehyde and oil, the effect of attachment time on the antifungal activity and the influence of oil concentration on the exosmosis rate of cells. This work is mainly to clarify the control and kill mechanism of cinnamon oil on the growth and cell integrity of fungi. Finally, the evaluations on the concentration changes of O_2_ and CO_2_, the decay and the activity of PPO (Polyphenylene Oxide) for the storage of sweet cherry are conducted in order to further validate the preservation mechanism of chitosan-oil coating. 

## 2. Materials and Methods

In this section, several types of sensor instruments, such as AFM, SEM (scanning electron microscope), GC-MS (gas chromatography-mass spectrometer) and gas analyzer, are used to clarify the preservation mechanism of chitosan film and cinnamon oil. AFM and SEM are two appropriate methods used to investigate the surface analysis of coating film. GC-MS is used to analyze the components in cinnamon oil. The methods including the antifungal activity transfer experiment and the comparison in exosmosis ratio of fungi cell are described. Finally, the analysis technologies for the O_2_ and CO_2_ concentrations, the decay rate and PPO activity are introduced. 

### 2.1. Preparation, AFM and SEM Observation of Chitosan Coating Film

In order to conduct the observation of the surface of the coating by AFM and SEM, the chitosan coating film was prepared according to the method reported by Xing et al. (2011a), Xing et al. (2015) and Sun et al. (2014) [3,4,38]. Different amounts of chitosan (0.25 g, 0.50 g and 1.0 g; Deacetylated ≥ 95%, viscosity ≤ 30 mPa·s; Ji’nan Haidebei Marline Bioengineering Co. Ltd., Ji’nan, China) were added into 100 mL distilled water with stirring by a magnetic stirrer. Then, acetic acid (0.50% v/v) and glycol (0.25% v/v) were added and stirred for 1 h in order to dissolve chitosan powder. After homogenized under aseptic conditions and stood for 2 h, 90 mL of the obtained chitosan solution was spread on a glass plate (20 cm × 20 cm). After being dried at room temperature for 4–5 days, they were peeled from the plate and conditioned at 4 °C prior to further observation by SEM and AFM [36].

The chitosan coating film was observed by SEM according to the method reported by Xing et al. (2015) [4]. The coating film with 1.0% chitosan was cut into thin pieces firstly and stuck on the sample stub. After Pt sputtering, the film piece was observed by SEM with the voltage of 5 kV acceleration.

The coating film with different concentration of chitosan was observed by AFM according to the method reported by Xing et al. (2011a,b) and Zdunek and Kurenda (2013) [3,9,39]. First, the chitosan coating film was cut into thin pieces (10 mm × 10 mm) using a small sharp knife and stuck on the stage. Ten pieces of film per treatment were scanned using the Tapping mode using a JSPM-5200 AFM (JEOL, Tokyo, Japan). In order to understand the roughness values *Ra* and *Rq*, the images were analyzed with Nanoscope software (Version 5.12, Tokyo, Japan). *Ra* and *Rq* are calculated as the following [40]:

$$R_{a}=\frac{1}{L_{x}L_{y}}\int_{0}^{L_{y}}\int_{0}^{L_{x}}\left|f(x,y)\right|dxdy$$

where *f(x,y)* is the surface relative to the center plane and *L_x_* and *L_y_* indicate the surface dimensions.

$$R_{q}=\sqrt{\frac{\sum {\left(Z_{i}-Z_{ave}\right)}^{2}}{N}}$$

where *Z_ave_* means the *Z* values average within the chosen area, *Z_i_* indicates the current *Z* value and *N* is the point number within the chosen area of samples. On the other hand, the thicknesses of chitosan coating films and the samples for AFM observation were also measured with a 0–25 mm dial thickness gauge with an accuracy of 0.01 mm in five random locations for one piece (n = 10 for each treatment). The thicknesses of film samples for AFM observation were 0.056 mm, 0.061 mm and 0.069 mm for films with the concentrations of chitosan at 0.25%, 0.50% and 1.0%, respectively. 

### 2.2. GC-MS Analysis of Cinnamon Oil

The GC-MS analysis technique for cinnamon oil was used according to method developed by Xing et al. (2010b) and Li et al. (2013) [26,41]. The GC-MS system used in this investigation has two components, which are Varian GC/MS 4000 equipped with a VF-5ms MS capillary column (30 m × 0.25 mm i.d. × 0.25 μm film; Scientific Instrument Services Inc., Varian, CA, USA), and an electron ionization system with ionization energy of 70 eV. He at the flow rate of 1 mL/min was used as the carrier gas during the analysis process. Firstly, the temperatures were set at 220 °C for injector and 280 °C for MS transfer line, respectively. Then, the temperature of the programmer was raised to 60–180 °C with a rate of 8 °C/min, which was held isothermal for 10 min and finally raised to 300 °C at the rate of 10 °C/min. Then, 1.0 μL diluted oil sample with hexane (1/10,000, v/v) was injected handy with the splitless mode. After the analysis procedure was completed, the identification of components in oil sample was conducted using literature data, the standard of NIST 05 by comparing their mass spectra and relative retention time.

### 2.3. Antifungal Activity of Trans-Cinnamaldehyde and Cinnamon Oil

The comparison on the antifungal activity between trans-cinnamaldehyde and cinnamon oil was determined against fungi including *P. citrinum* and *A. flavus* using the disk diffusion method as developed by Xing et al. (2011a) and Xing et al. (2012) [3,42]. First, the surfaces of potato dextrose-agar (PDA) plate were spread with 100 μL of inoculums containing 10^4^–10^5^ conidia/mL of *P. citrinum* and *A. flavus*. Then, the discs (*D* = 10 mm) impregnated with 10 μL trans-cinnamaldehyde and cinnamon oil with the concentrations of 100, 200, 300 and 400 μL/L were put on the center of PDA medium prepared above. Each treatment was performed in triplicate. The diameter of inhibition zones were measured after the plates incubated at 28 °C for 3–5 days.

### 2.4. Antifungal Activity Transfer Experiments of Cinnamon Oil

The method on the transfer experiments of antifungal activity for cinnamon oil against *P. citrinum* and *A. flavus* was developed from the investigation of Feng et al. (2006), Xing et al. (2011a) and Wang et al. (2012) with some modifications [3,43,44]. Certain volumes of cinnamon oil were added to PDA medium at the temperature of 40 °C in order to adjust the oil concentrations to 100, 200, 300 or 400 μL/L. The obtained mediums were shaken sufficiently in order to mix well and then prepared for use. Meanwhile, the PDA plates cultured for 7 days (*D* = 6 mm) with fungi *P. citrinum* and *A. flavus* were picked up with a sterile hole puncher and then put into the center of PDA plates containing cinnamon oil as prepared above. After contact for 0, 1, 3, 5, or 7 days, the PDA plate (*D* = 6 mm) was transferred to the center of newly PDA plates without cinnamon oil. The treated samples were performed in quintuplicate and cultured for 7 days into a 28 °C incubator. The diameters of colonies were measured and expressed in millimeters.

### 2.5. Effect of Different Oil Concentration on the Exosmosis Rate of Fungi Cell

Effects of different oil concentration on the exosmosis rate of fungi cell were conducted with the method developed from the investigation of Feng (2006) and Diao et al. (2014) with modifications [44,45]. First, cinnamon oil was added into the solution containing 0.05% Tween-20 and emulsified for 1 h before use. Then, the spores of *P. citrinum* and *A. flavus* were drawn from a slant PDA medium with inoculation loop and transferred to PDB medium. The cell concentrations in the medium were adjusted to about 5.0 × 10^7^ conidia/mL. The obtained PDB mediums containing *P. citrinum* and *A. flavus* spores were dispensed into flask and then the cinnamon oil emulsion prepared above was added. The final concentrations of cinnamon oil in the PDB mediums were adjusted to 0, 100, 200, 300 and 400 μL/L. The obtained flasks were put into a constant temperature shaker at 28 °C and 180 r/min. 

The flasks were picked up and centrifuged at 4 °C and 4000 g for 15 min after being cultured for 14 days. The obtained precipitate was washed with deionized water 1–2 times and was divided into two groups after being weighed. The samples were weighed and divided into a small beaker. The 20 mL deionized water was added in order to immerse the sample. The samples of Group A were placed in a vacuum oven with a vacuum gas drainage repeated 3–4 times. Then, the pressure was controlled at 450–500 mm Hg and the vacuum infiltration was restored to normal pressure after 30 min. The treated samples were shaken at 25–30 °C temperature for another 2–3 h. The samples of Group B were placed in a boiling water bath and heated for 30 min in order to destroy the plasma membrane completely. Finally, the extravasation fluids for these two treatments were poured into a small beaker and the conductivity was measured with electric conductivity meter. Each treatment was performed in quintuplicate. The relative electrolyte leakage rate = (the extravasation fluid conductivity of Group A/the extravasation fluid conductivity of Group B) × 100%.

### 2.6. Morphology of Normal Cell and Cell at the Edge of Inhibition Zone of A. flavus Observed by SEM 

Fungi cells of *A. flavus* on the edge of inhibition zone and on the control plate were picked up with the sterile hole puncher for observation. First, the fungi cell samples were fixed with 2% (v/v) glutaraldehyde at 4 °C for 12–18 h. After washing three times with phosphate buffer saline (PBS), the cells were post-fixed with 1% (w/v) osmium tetroxide prepared with 0.1 M PBS and dehydrated in a graded ethanol series from 30%, 50%, 70%, and 95% to absolute ethanol. Then, the dehydrated cells were subjected to critical point drying with liquid CO_2_. After Pt sputtering, the samples were observed by SEM equipped with a LEO Gemini 1530 field emission at a voltage of 5 kV acceleration [3,43]. 

### 2.7. Coating and Fruit Samples Preparation, O_2_ and CO_2_ Concentrations and Fruit Decay Analysis 

The chitosan-oil coating was prepared according to the method developed from the investigation of Xing et al. (2011a) and Xing et al. (2015) [3,4]. One gram of chitosan powder was added into 100 mL distilled water during stirring with a magnetic stirrer. The substances of acetic acid (0.50% v/v) and glycol (0.25% v/v) were also added and stirred for 1 h in order to dissolve chitosan in the water. After that, the compounds of cinnamon oil (0 and 0.10%) (Xianghui Bio-technology Co. Ltd., Shanghai, China) and Tween 80 (0.15%) were added and stirred for another 1 h. The final solution was homogenized under aseptic conditions and let stand for 2 h before use. 

“Brooks” sweet cherry fruits (*Prunus avium* L.) were harvested at the maturity stage from fruits base of MaoXian in Aba Tibetan and Qiang Autonomous Prefecture, China. Fruits were visually inspected as free of blemishes and defects, and then were precooled at 8 °C for 16–24 h. After precooling, fruits were divided into four groups with 200 fruits for each treatment. Four groups included the treatments of the chitosan (1.0%) + oil (0.10%) coating, the chitosan coating (1.0%), the cinnamon oil (0.10%) and the distilled water (control). Fruits were dipped into the different solutions for 5 min. The treated fruits were packaged with a polypropylene film (800 mm × 1000 mm) after kept over a plastic sieve for 120 min. All treatments were performed in triplicate and stored at 8 °C with the relative humidity of 85%–95% for analysis [3,4,9]. 

Concentrations of O_2_ and CO_2_ inside the packages were determined using an Oxygen and Carbon Dioxide gas analyzer (Shenzhen Empaer Technology Co. Ltd., Shenzhen, China) [9,46]. The decay rate of fruits in the packages was examined and calculated as the percentage of the weight of decay fruits to the total weight of fruits. PPO activity was assayed spectrophotometrically by the method based on Xing et al. (2015). Five grams of fruit tissue was homogenized with 10 mL extraction buffer (4 °C, 0.2 mol/L, pH 6.8 sodium phosphate buffer) in an external ice bath for 3 min including 0.1% polyvinylpolypyrrolidone. The homogenates were centrifuged for 12 min at 12,000 g and the supernatants were collected. The reaction solution was consisted of 0.1 mL crude extract and 2.9 mL substrate solution (0.02 mol/L catechol in 0.05 mol/L phosphate buffer, pH 6.5). An enzyme activity unit was defined as an increase of 0.0001 in absorbance per minute. The rates of catechol oxidation were examined at 420 nm for 2 min for all the treatment.

### 2.8. Statistical Analysis

Experimental data were analyzed by SPSS 16.0 software (SPSS Inc.) and reported as the mean ± S.D. The analysis of thickness and AFM for chitosan coating film was performed in decuplicate (10 pieces for each treatment). Effects of attachment time on the antifungal activity and the oil influence on the exosmosis rate of cells were conducted in quintuplicate. Other treatments were conducted in triplicate for each. The significant differences among the treatments were determined by the one way analysis of variance method followed by Student-Newman-Keul test.

## 3. Results and Discussion

In this section, the surface microstructure and roughness of chitosan coting is observed by AFM in order to clear its mechanism of slowing the respiration rate of fruits and its capacity as the carrier for cinnamon oil. Moreover, research work also needs to be conducted in order to clarify whether the antifungal activity of oil might be due to its main component. The effect of oil on the morphology change of cell is also discussed by comparing the exosmosis ratio of tested fungi cell before and after oil treatment and SEM observation. Finally, the functions on slowing the respiration rate, controlling the decay and PPO activity of fruits are investigated by in vivo tests and discussed in order to verify the preservation mechanism.

### 3.1. Morphological Observation of Chitosan Coating by SEM and AFM

**Figure 1:**
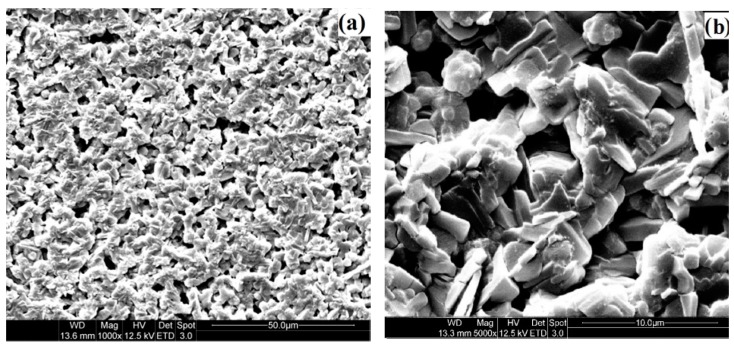
Morphological observation of chitosan coating (1.0%) by SEM: (**a**) 1000×; and (**b**) 5000×.

**Figure 2:**
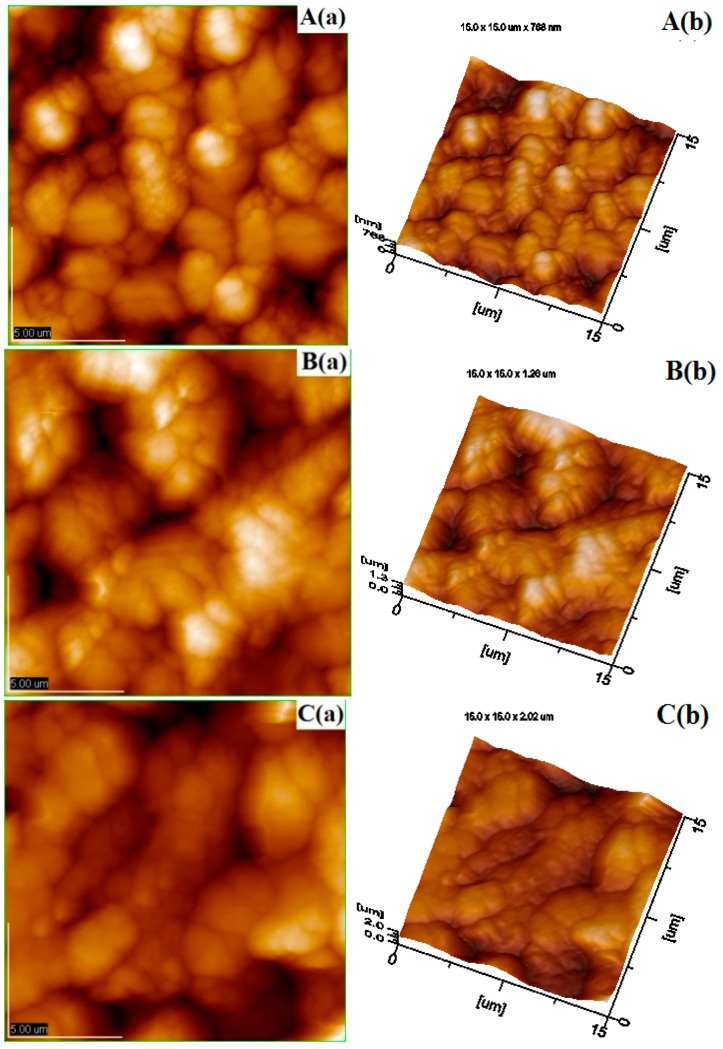
AFM analysis images for the morphology of chitosan coating (15 μm × 15 μm): (**A**) film with chitosan at 0.25%; (**B**) film with chitosan at 0.50%; and (**C**) film with chitosan at 1.0%, (a) AFM plane profile of chitosan film and (b) AFM three-dimensional profile of chitosan film.

**Figure 3:**
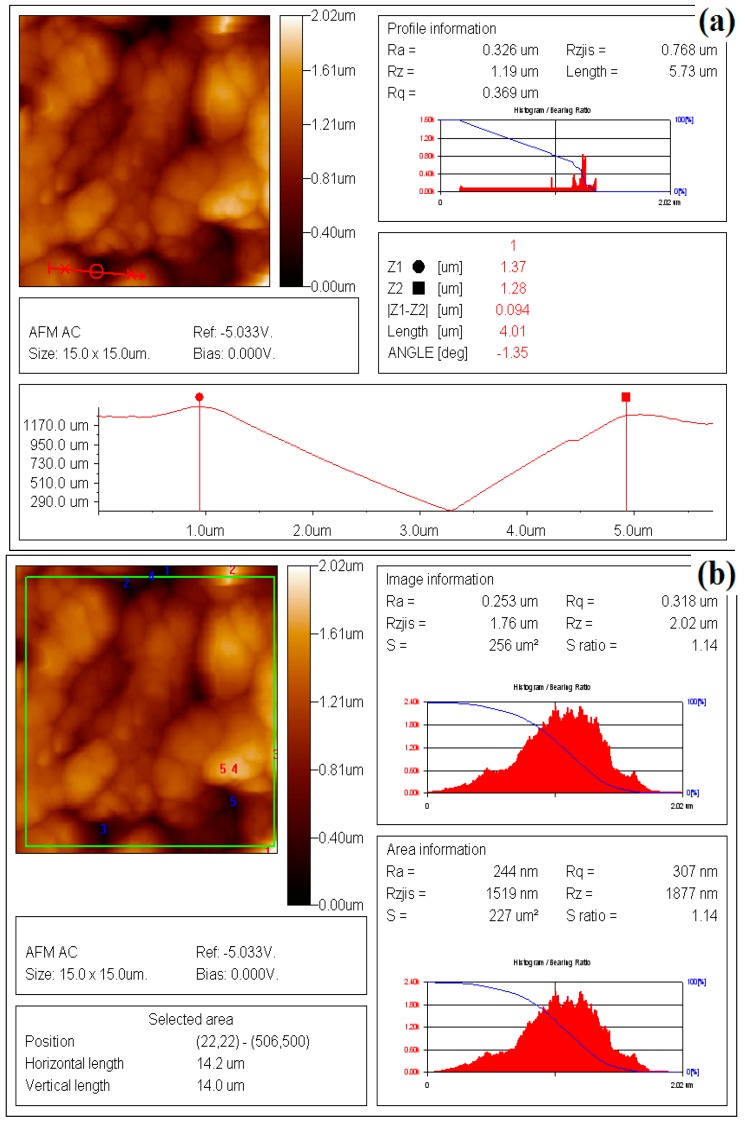
The sample images for the roughness of chitosan coating films analyzed by AFM: (**a**) AFM profile information of chitosan at 1.0%; and (**b**) AFM image information of chitosan at 1.0%.

In order to understand the mechanism and the oil loading capacity of edible coatings, morphology of chitosan coating film was observed using SEM and AFM. Many micropores are found on the chitosan film (1.0%) from the results of SEM observation in Figure 1. The plane and the three-dimensional profiles of coating film with different concentration of chitosan are shown in Figure 2. The morphology of AFM also illustrates that many micropores are observed on the chitosan coating film. Based on the AFM analysis on the 10 pieces of chitosan film for each concentration, the total number of micropores (10 pieces together) on the surface of selected area film decreases from 86 to 53 with increasing the concentration of chitosan from 0.25% to 1.0% (data are not listed in table form). The micropores on the surface of coating film with chitosan at 1.0% could also be validated from SEM findings, as shown in Figure 1. The distributions of micropores on the surface of film were not consistent among those prepared with different concentration of chitosan, which could also affect the smoothness and roughness of the coating surface. Thus, the parameters of surface roughness for chitosan coating membrane were also analyzed by AFM and expressed in terms of *Ra* and *Rq* of chitosan membrane surface. These two roughness parameters, *Ra* and *Rq*, indicated the mean roughness and the root mean square of *Z* data, respectively, are calculated from sample mathematical expressions and illustrated in Table 1. The sample images of analysis on the *Ra* and *Rq* of coating films with chitosan at 1.0% are shown in Figure 3a,b. As shown in Table 1, in the micropores profile information, the *Ra* and *Rq* of coating film with chitosan at 0.25% are 0.244 μm and 0.289 μm, respectively, which increase to 0.399 μm and 0.449 μm for the coating film with chitosan at 1.0%. Furthermore, in the image information analysis, the *Ra* and *Rq* of coating film with chitosan at 0.25% are 0.131 μm and 0.159 μm, which increase to 0.247 μm and 0.303 μm for the film containing chitosan at the concentration of 1.0%, respectively. The roughness of chitosan coating films could be improved by increasing the concentration of chitosan. Significance differences among the roughness of coating film surfaces are observed from the results among different concentrations of chitosan films. On the other hand, the thicknesses of films are measured and shown in Table 1. The thickness of films varied from 0.056 mm to 0.068 mm with increasing the chitosan concentration from 0.25% to 1.0%. However, no significant difference is found between films prepared with 0.25% and 0.50% chitosan. Differences in the thickness of coating film could be attributed to the different concentrations of chitosan added in the film-form solution. 

The roughness of coating film is an inherent property of chitosan-based polymer in which chitosan forms the coating film with micro-perforated structure and influences the smoothness of fruit surfaces. Results in AFM images indicate that the chitosan coating membrane with many micropores could be used as the carrier of active compounds, which might fill the empty spaces of surface micropores [3,9,47]. Moreover, the chitosan coating on the surface of fruits might induce the increase in the concentration of CO_2_ and the decrease in the levels of O_2_ in the package, which could influence the respiration rate of fruits [6,9]. Moreover, it has the potential for inducing phenolic contents and defense-related enzymes such as PPO in fruit products [9,14]. These results are consisted with the investigations conducted by Xing et al. (2010a), Xing et al. (2011a) and Xing et al. (2015) [3,4,18]. As reported by Xing et al. (2010a), the optimum concentrations of O_2_ and CO_2_ in packages of fresh-cut lotus root were created by the application of chitosan coating. Furthermore, the inhomogeneous distribution of micropores is found on the surface of film with different concentration of chitosan. The number of micropores in the film surface might decrease with increasing the addition of chitosan in the coating solution system. This might be due the interaction among the water, emulsifier, acetic acid, glycol and the increased chitosan in the system. Moreover, the inhomogeneous distribution of micropores, meaning the uneven distribution of the peaks and valleys in the chitosan coating, could significantly affect the *Ra* and *Rq* values of film surface [48,49,50,51,52,53,54]. The surfaces with fewer micropores could become less sharp and show a smoother appearance, which might also be influenced by the added concentration of chitosan. The surface of film becomes smoother with the higher addition of chitosan. The good uniform microstructure of chitosan coating films are observed from SEM images, which indicates that the mixtures of chitosan, acetic acid and glycerol are homogenous in the coating film. These results could be explained by the interaction of the higher amount chitosan with other components in the prepared system, the adsorbed action of surface tension for coating liquid and the different number of micropores formed on the film surface [49,50,51,52]. Xing et al. (2011b) also reported that the roughness value of control group was higher than the chitosan–oil coated group fruits [9]. However, as reported by Segota et al. (2012), the small amount addition of polyethylene glycol to TiO_2_ film could drastically influence the roughness parameters. This might be due to the different property of added substances and based film. Furthermore, the thickness of films could be affected by the addition of chitosan, the type of chitosan used and the temperature of drying [38,55]. The addition of a relatively lower dose of chitosan exhibited the lower thickness. As reported by Sun et al. (2014), differences in the thickness of chitosan-Gallic acid films might be due to the addition and interaction of chitosan and Gallic acid [38]. The roughness and thickness of film surfaces are also influenced by the different chosen area, the analysis method and the film materials. Thus, chitosan-based solution coated on fruits might exhibit the difference surface characteristics compared to the film prepared from the dried solution. The influence of chitosan addition on the surface characteristics of films could be directly observed from the analysis results of film obtained from the dried solution, which is the in vitro analysis method used in many similar investigations [38,56,57,58]. After being coated on fruits, the ordered microstructure of film may be disrupted by the different types of fruit surfaces, which also affects the number/size and disruption of micropores and further influence the thickness and gas permeability of coating film covered on the surface of fruits. The optimum concentrations of O_2_ and CO_2_ in the packages could be created by the application of coating with the suitable thickness and chitosan concentration [18]. However, a coating with higher thickness or more chitosan might induce anaerobic respiration and quicken the senescence of fruits. Water vapor permeability and oxygen permeability of coating film might also be influenced by the addition and interaction of cinnamon oil and surfactant in the film-forming dispersion [38,56,57,58]. A coating film with a suitable number/size of micropores could form a protective barrier to reduce decay, water loss and respiration rate of fruit, which might also influence the resistance of tissue, the antioxidant enzyme activities and the senescence process of fruits [4,9,58,59]. These synergetic functions and mechanisms still need to be further investigated and clearly defined.

### 3.2. Chemical Composition of Cinnamon Oil Analyzed by GC-MS

**Figure 4:**
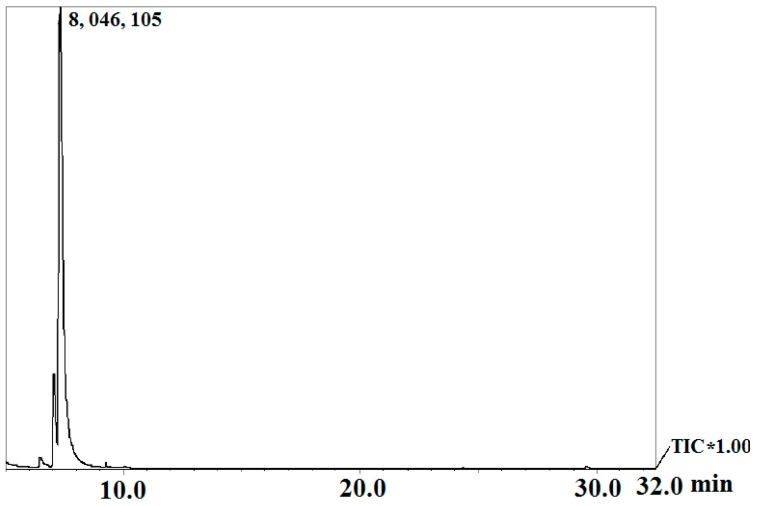
The total ion current for GC-MS analysis on the components of cinnamon oil.

In order to clear the main components, the analysis of the chemical composition of cinnamon oil was conducted by GC-MS. The chemical consistent of cinnamon oil is listed in Table 2. The total ion current of GC-MS analysis can be seen in Figure 4. As shown in Table 2, a total of 17 components are identified in the cinnamon oil sample. Results indicated that the most abundant component is trans-cinnamaldehyde, which is 85.64% of the relative percentage in cinnamon oil. In addition, cinnamon oil also contains cinnamaldehyde (1.37%) and benzenepropanol, a-methyl- (11.43%). The area percentages of other volatile compounds identified by GC-MS are less than 1.0%. The volatile compounds are comprised of two aldehydes, six ethers, two alcohols and seven others. From the analysis result of GC-MS, it could be confirmed that trans-cinnamaldehyde is the major component with the highest area percentage in the tested cinnamon oil. 

Natural active compounds such as cinnamon oil should be developed because that the exceeded residue of chemical fungicides could cause an increased risk of diseases and increased probability of affecting the human health [4,25,26,27,28,41,60]. The component identification is very important for the application of cinnamon oil, which is a high quality natural antimicrobial agent and widely used in the storage of fruits. The result in this work indicates that the compound trans-cinnamaldehyde should be the major component in cinnamon oil. Li et al. (2013) also indicated that trans-cinnamaldehyde was the main compound of nine cinnamon bark oils using GC-MS analysis [41]. However, differences in the main compounds of cinnamon oil have been observed by other researchers. The investigations of Wang et al. (2005), Xing et al. (2010b) and Kim et al. (2015) indicated that the major compound in cinnamon oil was cinnamaldehyde [26,27,61]. The component of cinnamaldehyde is also identified in this research, as only 1.37% of the relative percentage in cinnamon oil. Moreover, Singh et al. (2007) have reported that eugenol is the major volatile compound (87.3%) and no trans-cinnamaldehyde was detected in the oil of *C. zeylanicum* leaf [62]. Goñi et al. (2009) expressed that the major component of cinnamon essential oils is trans-cinnamaldehyde or cinnamaldehyde [63]. Todd et al. (2013) and Kaskatepe et al. (2016) indicated that the major substance of Cinnamon was cinnamaldehyde [64,65]. This difference might be due to the spices of cinnamon bark, the environmental factors, the origin and parts of cinnamon, the analysis method and other factors [25,26,27,66,67,68]. More importantly, according to the results reported by Wang et al. (2005), Xing et al. (2010b) and Xing et al. (2011a), cinnamon oil containing cinnamaldehyde and/or trans-cinnamaldehyde exhibits an excellent antimicrobial property against fruits pathogens including bacterial and fungi [3,26,61]. Thus, it is suspected that the main components of trans-cinnamaldehyde and/or cinnamaldehyde are the principal antifungal agent in cinnamon oil. Further research on this point is discussed in the next section. 

### 3.3. In vitro Antifungal Activity of Cinnamon Oil and Trans-Cinnamaldehyde against P. citrinum and A. flavus

**Figure 5:**
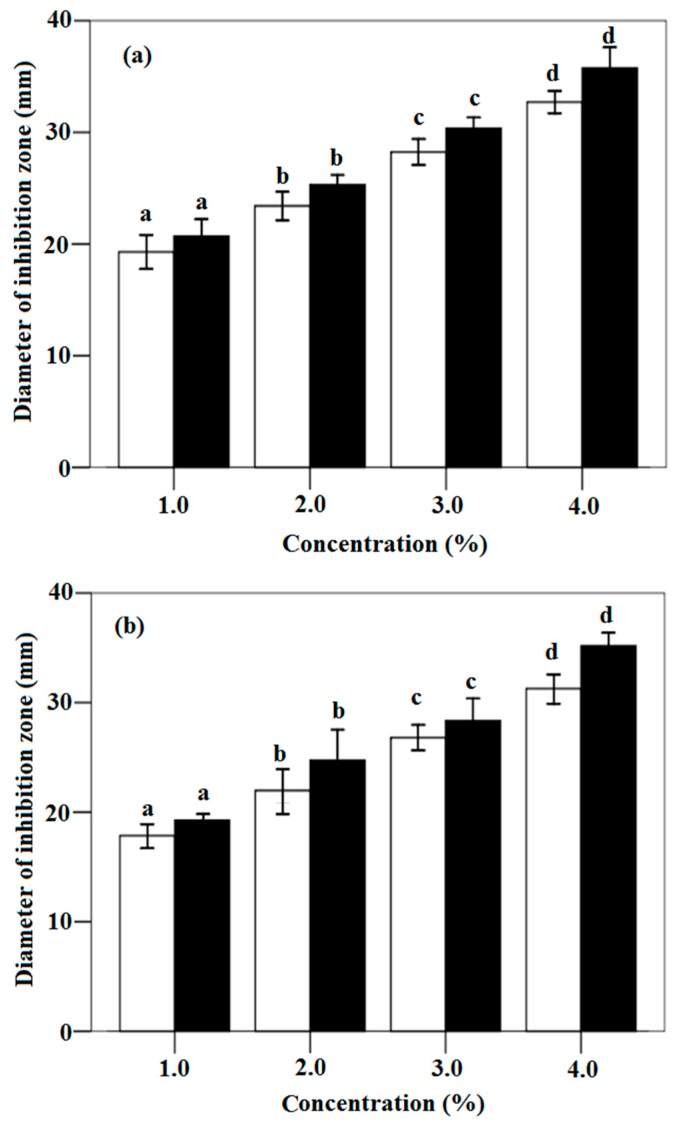
The antimicrobial activity of trans-cinnamaldehydes and cinnamon oil against: *P. citrinum* (**a**); and *A. flavus* (**b**). □: trans-cinnamaldehydes; ■: cinnamon oil; mean bars with different letters (a–d) differ significantly at *p* < 0.05.

In order to validate whether the antifungal property of cinnamon oil is due to its main component, the in vitro activities against two fungi, *P. citrinum* and *A. flavus*, were compared between trans-cinnamaldehyde and cinnamon oil. Results in Figure 5a indicate that the diameters of inhibition zones for trans-cinnamaldehyde and cinnamon oil against *P. citrinum* improve from 19.17 mm to 33.17 mm and from 20.81 mm to 36.23 mm with increasing the concentration from 1.0% to 4.0%, respectively. A similar result is also observed against *A. flavus* in Figure 5b: the zone diameters improve from 17.93 mm and 19.07 mm to 31.12 mm and 35.28 mm for the increase of the concentration of trans-cinnamaldehyde and cinnamon oil from 1.0% to 4.0%, respectively. However, significant differences are found in the antifungal activity of cinnamon oil and trans-cinnamaldehyde among different concentrations from the analysis result in Figure 5a,b. This means the inhibitory effects of trans-cinnamaldehyde and cinnamon oil are proportional to the concentration of trans-cinnamaldehyde. Excellent antifungal activities of oil and trans-cinnamaldehyde are found. Their antifungal activities against the growth of *P. citrinum* are higher than that against *A. flavus*. 

These results shows that the killing activity of oil samples against fungi should be mainly due to its main component trans-cinnamaldehyde [26,27,43,61]. The antifungal activity of chitosan-based coating could enhance by the addition of cinnamon oil. These results were also reported by Kanatt et al. (2008), Xing et al. (2010b) and Xing et al. (2011a) [3,13,26]. Xing et al. (2010b) had observed that the strong inhibitory effect of cinnamon oil against all the tested microorganisms should be due to its main component cinnamaldehyde [9,26,27]. On the other hand, result in this work indicates that significant improvement in the inhibition on fungi growth was observed, which might be due to the increased levels of trans-cinnamaldehyde or cinnamon oil used. The antifungal activity of trans-cinnamaldehyde is only a little lower than that of cinnamon oil. It can be found that trans-cinnamaldehyde is the most effective components in cinnamon oil for inhibiting the fungi growth. More importantly, the higher antifungal activity of cinnamon oil should be due to the synergistic action of trans-cinnamaldehyde, cinnamaldehyde, and other components (such as benzenepropanolb, a-methyl-; propanoic acid, 2-methyl-, and 3-phenylpropyl ester). The inhibition of trans-cinnamaldehyde and cinnamaldehyde on the synthesizing enzymes of fungal cell was also found [3,26,37,61,64]. Cinnamaldehyde with a strong antifungal activity was reported by Wang et al. (2005) [61]. According to the investigation of Lambert et al. (2001) and Todd et al. (2013), the chemical constituents in oils could accumulate in the lipid-rich environments of cell membrane structures and cause its functional damage [64,68]. Clemente et al. (2016) demonstrated that the main bacterial target of cinnamaldehyde as the major component of cinnamon oil could cross the cell wall and act by the interaction of cinnamaldehyde carbonyl group and proteins [37,64]. It could be concluded that the antifungal activity might be due to the major and minor components in cinnamon oil, which means synergies of all components in cinnamon oil [26,41,43,47]. Burt (2004) has reported that the minor components might be critical to the activity and could provide a synergistic effect by the interactions among active substances in oil [69,70,71]. These findings emphasize the killing effectiveness of cinnamon oil and its principle component trans-cinnamaldehyde, which might induce the irreversible deleterious structural and morphological alterations of fungi cells [43,47]. 

### 3.4. Antifungal Activity of Cinnamon Oil for Different Attachment Time against P. citrinum and A. flavus

**Figure 6:**
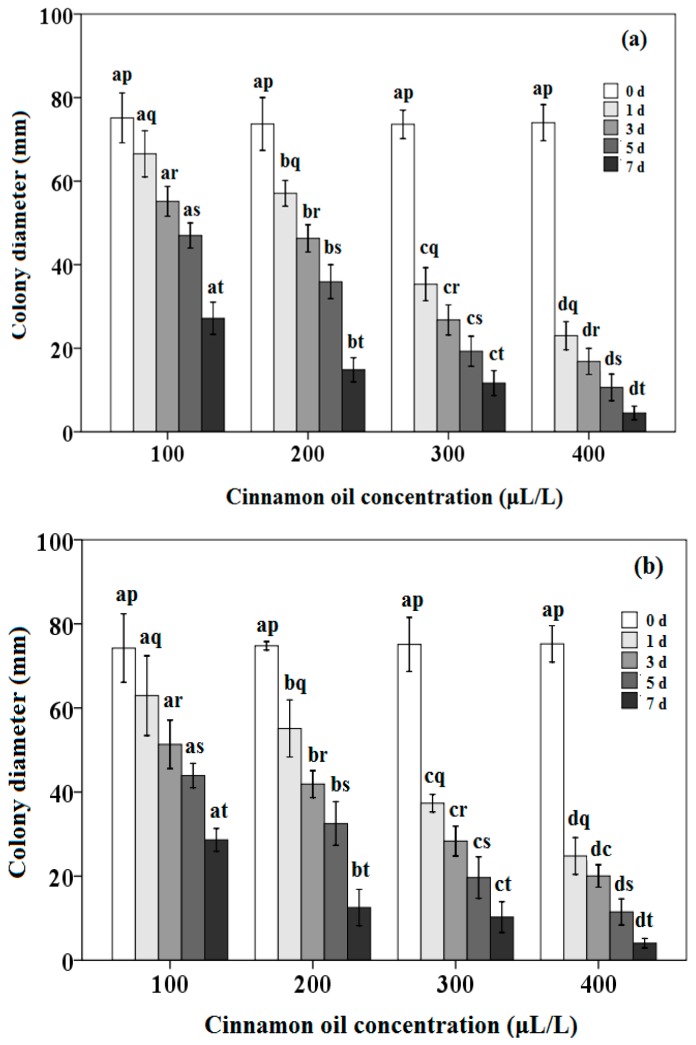
Antifungal activity of different attachment time of cinnamon oil with different concentrations against: *P. citrinum* (**a**); and *A. flavus* (**b**). Mean bars with different letters (a–d) indicate significant differences at *p* < 0.05 for the different concentration of cinnamon oil at the same attachment time. Mean bars with different letters (p–t) indicate significant differences at *p* < 0.05 for the different attachment time at the same concentration of cinnamon oil.

In order to investigate the mechanism for antifungal activity, the colony diameters of fungi growth after treated by cinnamon oil for different attachment time were examined. The index indicates impaired growth, and is used to describe the antimicrobial activity of cinnamon oil on the growth of *P. citrinum* and *A. flavus* on the PDA after the original fungi cell exposed to the active cinnamon oil with different time. As shown in Figure 6a, for the inhibition activity of oil against *P. citrinum*, the colony diameters decreased from 75.14 mm to 27.18 mm and from 74.01 mm to 4.49 mm with increasing the attachment time from 0 to 7 days at the concentration of cinnamon oil 100 μL/L and 400 μL/L, respectively. On the other hand, as can be seen in Figure 6b, with the attachment time of cinnamon oil on the fungi cells from 0 to 7 days, the colony diameters of *A. flavus* also decreased from 74.24 mm to 28.64 mm and from 75.22 mm to 4.04 mm with the oil concentration at 100 μL/L and 400 μL/L, respectively. Cinnamon oil almost inhibited the mycelial and spore growth of *P. citrinum* and *A. flavus* with the attached treatment of cinnamon oil at the concentration of 400 μL/L for 7 days. Furthermore, as can be seen in Figure 6, differences in the antifungal activity were observed among different attachment time for 0 to 7 days of cinnamon oil at the same oil concentration against *P. citrinum* and *A. flavus*. For the antifungal activity of cinnamon oil with the same attachment time from 1 to 7 days, there were differences among the concentrations of oil from 100 μL/L to 400 μL/L. However, no difference could be seen in the attachment time of 0 day among different oil concentration. 

These results illustrate that the inhibition on the growth of *P. citrinum* and *A. flavus* could be affected by the attachment time and the concentration of cinnamon oil [3,26,43]. Cinnamon oil with the lower concentration could only cause the delay of conidia germination [3,43,71,72]. The longer attachment time and higher concentration of cinnamon oil could exhibit the higher inhibition on the growth and the higher kill activity of these two tested fungi. The most fungi cells could be inhibited or killed with the oil concentration at 400 μL/L for 7 days of the attachment time. The shorter attachment time and the lower concentration of active compounds could affect the initial development of molds, and produce a delay in the growth [3,67,71]. These results suggest a fungicidal effect at higher concentrations (400 μL/L) and an inhibition effect of cinnamon oil at lower concentrations (100 μL/L). For the durability of antifungal activity, the inhibition could be maintained for 7 days, especially for the higher concentration of oil. This might be due to these two reasons: the incomplete formation of conidia and the damage to the conidia caused by oil after the conidiophore formed [3,26,66,71]. The active paper disc created an inhibition area where the growth of fungi was inhibited, a clear zone where only white mycelium was presented and a strong inhibition in sporulation was noticed. These evident morphological changes can be appreciated due to the exposure to the vapor phase of oil during the attachment period. Volatile compounds in oil could induce the abnormalities in the cell wall structure, the disruption of hyphae, the loss of cytoplasm, the decrease in the conidia number and the conidiophores development, thereby disrupting various metabolic activities [2,71,72,73,74,75]. On the other hand, differences in the antifungal activity among different attachment time of oil with the same oil concentration might be due to the sustained release and action of oil vapor phase from the liquid phase [36,37,63]. The difference of the antifungal activity among different oil concentrations with the same attachment time might be due to the different concentration of oil vapor phase released from liquid phase and thereby inducing different killing or inhibiting actions [36,37,63]. 

### 3.5. Effect of Cinnamon Oil on the Exosmosis Ratio of P. citrinum and A. flavus 

**Figure 7:**
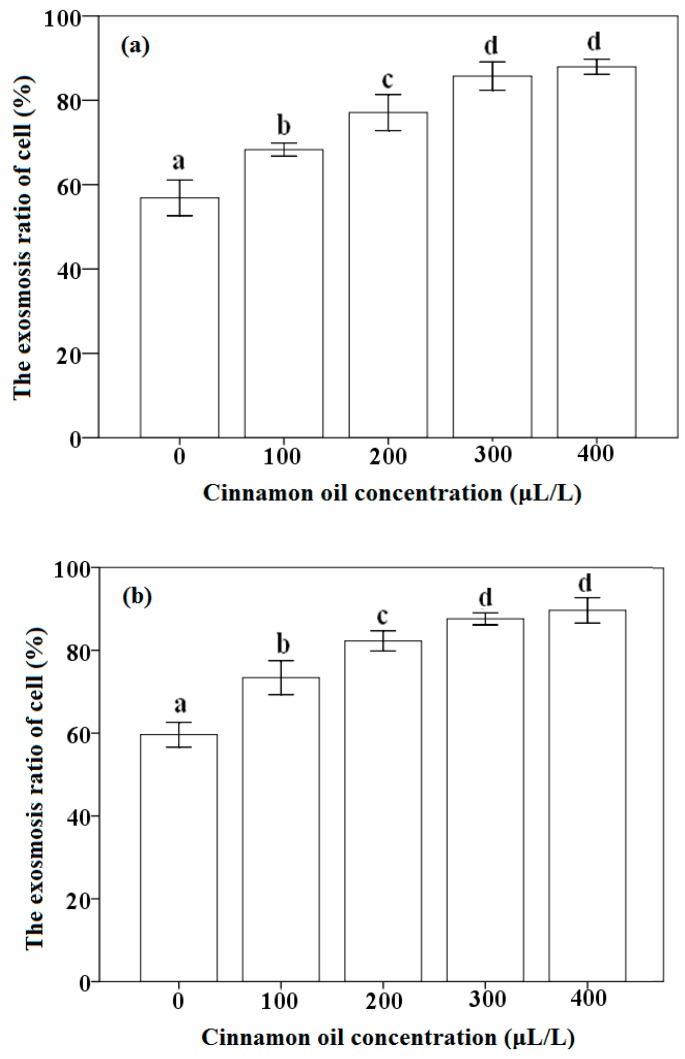
Effect of the concentration of cinnamon oil on the exosmosis ratio of: *P. citrinum* (**a**); and *A. flavus* (**b**). Mean bars with different letters (a–d) differ significantly at *p* < 0.05.

**Figure 8:**
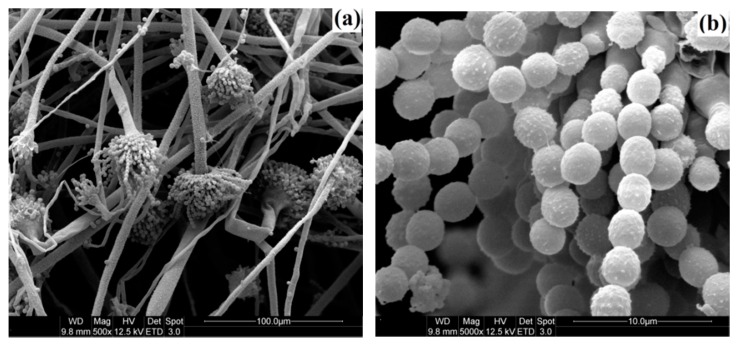

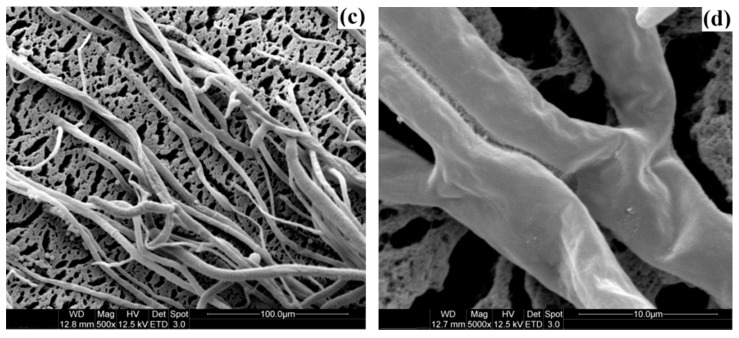
SEM images of normal cells ((**a**) 500×; and (**b**) 5000×)) and cells on the edge of inhibition zone ((**c**) 500×; and (**d**) 5000×) of *A. flavus*.

The effects of different concentration of cinnamon oil on the exosmosis ratio of *P. citrinum* and *A. flavus* were investigated. Results in Figure 7a,b show that the exosmosis ratios of fungal cells with the concentration of cinnamon oil tests exhibit a close agreement with those above results of antifungal activity. The exosmosis ratios for the cell of *P. citrinum* and *A. flavus* as the control are 56.85% and 59.61%, respectively. However, the exosmosis ratios improve from 68.31% and 73.39% to 87.95% and 89.65% for *P. citrinum* and *A. flavus* with increasing the concentration from 100 μL/L to 400 μL/L, respectively. An increase in the exosmosis ratio of fungal cell is observed with improving the cinnamon oil concentration. Moreover, in order to investigate the disruption mechanism of cinnamon oil on the morphological structure of fungi cell, using *A. flavus* as an example, the morphological observation of cell around the inhibition zone and the normal fungi cell were conducted by SEM. As can be seen in Figure 8a,b, the normal cell of *A. flavus* on PDA shows a normal morphology with formed spores and linearly shaped hyphae. The hyphae and spore of normal *A. flavus* appear regular and homogeneous with the smooth walls. However, results in Figure 8c,d indicate that the mycelium of *A. flavus* with wizened, irregular and atrophied shapes is observed in the cells around the inhibition zone. From the images of SEM, it could be found that the phenomena of hyphae with shrank, slender and winding and the loss of the linearity on the hyphal surface were observed. The spore growth of *A. flavus* was inhibited completely by cinnamon oil. The disruption and control action of cinnamon oil might occur on the development of the spore and hypha of fungi cells, which should be due to the strong antimicrobial action of components in cinnamon oil, such as trans-cinnamaldehyde and cinnamaldehyde.

The investigations on the exosmosis ratio of fungi cells treated with different oil concentration and on the morphological observation of fungi cell could helpful to understand the mechanism of antifungal activity. The enhanced exosmosis ratio of fungi cells is related to the action and the increasing concentration of cinnamon oil. The disruption mechanism should be because most of the hypha cell walls became irregular and the contents either disappeared or were denatured. These also suggest that the oil might penetrate through the cell walls and membranes, causing mechanical damage and disrupting the cell metabolism [41,71]. SEM analysis also indicates that after the oil acted on *A. flavus* cells, the growth of spores and mycelium were destroyed and inhibited [26]. These processes could further damage the organelles and cell nucleus, and allow the contents to escape from the cells, eventually resulting in a cell burst and then death [36,37,41,71]. The investigation of Tzortzakis (2009) also indicated that the effects of cinnamon oil reduced the germination of spore and the growth of germ tube in *Colletotrichum coccodes*, *Botrytis cinerea* and *Rhizopus stolonifer* was mainly dependent on its concentration used [76]. The result reported by other workers also demonstrated that cinnamaldehyde could pass the cytoplasm and translocate to the cell nucleus of fungi, and then interfere with the synthesis of protein and RNA in the cell [66,77]. It is also mentioned that the hydroxyl groups in active components could affect the biosynthesis of mycotoxins by forming the hydrogen bonds with active enzymes and thereby resulting in deactivation [3,26,56,78]. 

### 3.6. Gas Composition in Packages and Fruits Decay Treated by Chitosan-Oil Coating 

**Figure 9:**
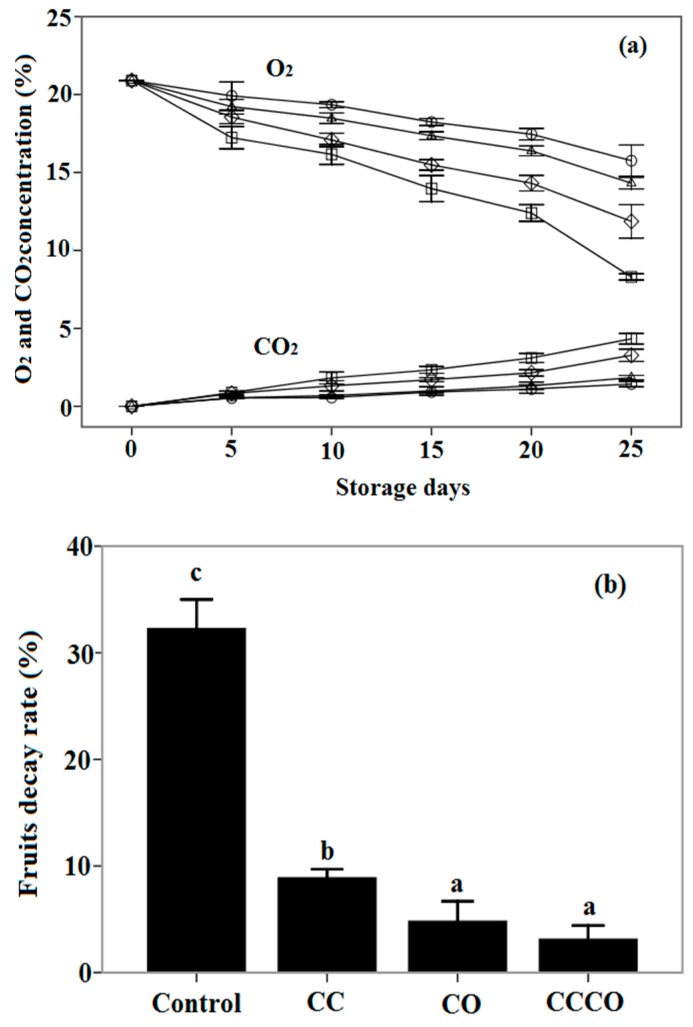

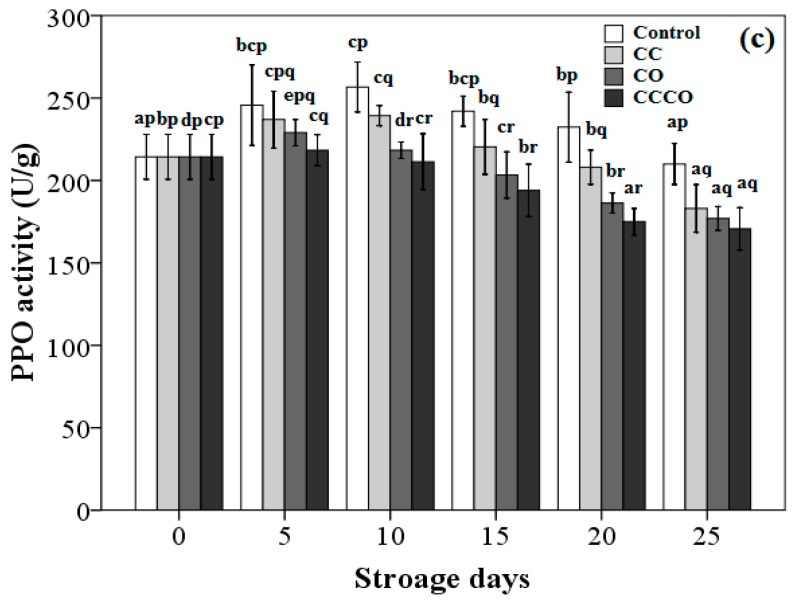
Effect of coating treatments on: the concentrations of O_2_ and CO_2_ in the packages (**a**); fruit decay (**b**); and PPO (Polyphenylene Oxide) activity of fruits (**c**). □: Control; △: Chitosan coating; ◇: 0.25% Cinnamon oil; ○: Chitosan coating containing cinnamon oil; CC: Chitosan Coating; CO: Cinnamon Oil, CCCO: Chitosan Coating containing Cinnamon oil. Mean bars with different letters (a–e) differ significantly among different storage days for the same treatment at *p* < 0.05. Mean bars with different letters (p–r) differ significantly among different treatments for the same storage days at *p* < 0.05.

Chitosan-oil coating as a barrier of gas and a carrier of the antimicrobial agent could create an internal modified atmosphere in the package and control the decay of fruits during the storage period [18]. The changes in the concentration of O_2_ and CO_2_ in the packages of sweet cherry fruits with different treatments were analyzed with gas sensors. As can be seen in Figure 9a, the O_2_ concentrations in the package for the samples treated by the chitosan coating with cinnamon oil and the chitosan coating are 15.76% and 14.32% after 25 days of storage, respectively. The O_2_ concentration inside the packages of control fruits was reduced significantly, and was only 8.3% for the control samples. On the other hand, the CO_2_ concentration in the packages for the treatments of chitosan-oil coating and control are 4.33% and 1.43%, respectively. The production of CO_2_ in the package for the chitosan-oil treatment are lower compared to that of fruits coated with only chitosan or only oil treatment. Moreover, during the storage time, the cheery fruits could be infected by molds bringing the fruits from the field, which could be strongly delayed by the treatment of chitosan-oil coating. As the results in Figure 9b show, the higher decay (32.37%) rate of uncoated cheery fruits is observed at the end of storage. However, cherry fruits coated with the chitosan-oil composite film display the lowest decay rate throughout the 25-day storage period, at 3.13%, which is much lower than that of control fruits. The decay rate of fruits treated by cinnamon oil is 4.83% after 25-day storage. The PPO activity of fruits treated with different coatings is shown in Figure 9c; the lowest activity of PPO was observed in the fruits treated with the chitosan-oil coating, which was 170.7 U/g on the 25th day of storage. However, the control fruits exhibited the highest activity for all of the storages. PPO activity is 210.0 U/g at the end of storage. It could be inhibited by the chitosan-oil coating during the storage period. Furthermore, as can be seen in Figure 9c, differences in PPO activity were observed among different storage days for 5 to 25 days of both treatments of only oil treatments and the chitosan-oil coating. Differences in the PPO activity of fruits were also found among the different treatment at the same storage days, from 5 to 25 days. The coating of chitosan with cinnamon oil could be used in maintaining the quality of cherry fruits. 

These results indicate that the respiration rate of sweet fruits reflected by the concentrations of O_2_ and CO_2_ was markedly affected by the treatments of chitosan-based coating during storage [4,9,10,18]. Results of the decrease in the levels of O_2_ and the increase in the concentration of CO_2_ in the packages denote that chitosan-based coating is beneficial for reducing the respiration rate of fruits in the package [2,9,10,18]. The chitosan-based coating with/without cinnamon oil plays an important role in the modification of gas concentration. Incorporation of cinnamon oil in the chitosan coating could reduce the O_2_ consumption of fruits and inhibit the activity of PPO compared with that of other treatments, especially for the control samples. This is possibly because of the antioxidant activity of cinnamon oil and the interaction between chitosan and cinnamon oil as the active substance, which is different from both the only chitosan coating and the only oil treatment [3,4,9,23,79]. Significant differences regarding the consumption of O_2_ and the production of CO_2_ are also found between the coated fruits and control samples at the end of storage. Our results are in agreement with the findings of other researchers: the coating treatment could reduce the consumption of O_2_ for strawberries [26,80,81,82,83]. Perdones et al. (2012) have reported that the gas permeability was influenced by the chitosan coatings on the fruits surface [58]. The equilibrium O_2_ and CO_2_ levels might be created by the barrier of chitosan-based films, which might benefit the storage quality of cherry fruits [84,85]. On the other hand, the control of postharvest decay in cherry fruits has been of interest because it could significantly affect the quality safety of fruits during storage [86,87,88,89,90,91]. Fruits coated by this composite coating exhibit lower percentages of decay than those of control samples, which might be because of the higher inhibition of the pathogen growth of cinnamon oil in the coating and also due to the higher levels of CO_2_ in the packages [3,9]. The antioxidant property of some compounds in cinnamon oil could also inhibit the PPO activity in fruits and be helpful for keeping the quality of produce [3,4,6,9]. The chitosan-based coating on the surface of cherry fruits could control the pathogen infection by reducing the diffusion of oil vapor and slow the physiological processes in fruits [3,4,6]. The mechanism of the induction of chitosan-oil coating on the defense-related enzymes, the permeability and integrity of fruit cell membrane, and odors in fruits should be further investigated [3,4,6,9,92,93]. 

## 4. Conclusions

Preservation mechanism of chitosan coating and cinnamon oil for fruits storage is very important for understanding its application effect. The analysis of AFM indicates that many micropores exist in the chitosan based coating. The antifungal property of cinnamon oil should be mainly due to its main constituent, trans-cinnamaldehyde. It is also proportional to the concentration of oil and the attachment time. Compared to the normal cell, the wizened mycelium of *A. flavus* around the inhibition zone is observed, and the growth of spores could be inhibited completely. These indicate that the preservation mechanism of chitosan-oil coating should be due to the micropores structure of chitosan coating as the carrier and the antifungal activity of oil, which could slow the respiration rate and control the decay of fruits during the storage period. The antioxidant mechanism of chitosan-based coating and cinnamon oil, such as the in vitro and in vivo antioxidant activity and mechanism of cinnamon oil, the influence mechanism of chitosan-oil coating on the defense-related enzymes, free radical scavenging activity, and the permeability and integrity of fruit cell membrane in fruits. These effects will need to be researched further and will be published in the future.

## Figures and Tables

**Table 1:** The arithmetic average roughness (*Ra*) and the root mean square roughness (*Rq*) of chitosan coating films analyzed by AFM. CC: Chitosan Concentration; MPCF: Micropore profile in chitosan film; IICF: Image information of chitosan film; CCFT: chitosan coating film thickness; Mean data with different letters (**a**–**d**) differ significantly at *p* < 0.05.

CC	Samples	*Ra* (μm)	*Rq* (μm)	CCFT (mm)
0.25%	MPCF	0.244 ^ab^ ± 0.042	0.289 ^b^ ± 0.034	0.056 ^a^ ± 0.004
	IICF	0.131^a^ ± 0.018	0.159 ^a^ ± 0.019	
0.50%	MPCF	0.295 ^b^ ± 0.051	0.334 ^b^ ± 0.050	0.061 ^a^ ± 0.004
	IICF	0.181^ab^ ± 0.029	0.228 ^ab^ ± 0.029	
1.0%	MPCF	0.399 ^c^ ± 0.093	0.449 ^c^ ± 0.106	0.068 ^b^ ± 0.006
	IICF	0.247 ^ab^ ± 0.050	0.303 ^b^ ± 0.017	

**Table 2:** The relative contents of chemical consistent in cinnamon oil analyzed by GC-MS.

Compounds	Retention Time (min)	Relative Percentage (%)
Cinnamaldehyde	6.489	1.37
Propanoic acid, 2-methyl-, 3-phenylpropyl ester	6.700	0.47
Benzenepropanol, a-methyl-	7.051	11.43
trans-Cinnamaldehyde	7.322	85.64
Cyclobutanone	8.767	0.02
1-Hexadecyl-2,3-dihydro-1*H*-indene	8.833	0.03
1-Chloropropane	8.992	0.02
Aziridine, 1-methyl-	9.025	0.01
2-Penten-1-ol,5-[(1*R*,3*R*,6*S*)-2,3-dimethyltricyclo[2.2.1.02,6]hept-3-yl]-2-methyl-, (2*Z*)-	9.258	0.24
zingiberene	9.477	0.05
1,2-Benzenedicarboxylic acid dimethyl ester	10.052	0.18
Propargyl propionate	11.849	0.01
Methyl pentanoate	22.292	0.04
1-(Aminooxy)-2-propene	22.892	0.03
Arachidic Acid Ethyl Ester	24.333	0.12
2-Cyclohexylethanol	29.375	0.05
9-Octadecenoic acid(9*Z*)-, ethyl ester	29.582	0.29
